# Unmasking the coronavirus pandemic impact on students’ academic performance (University of Cape Coast—Ghana)

**DOI:** 10.1371/journal.pgph.0003409

**Published:** 2024-07-16

**Authors:** Jonathan Kissi, Wisdom Wise Kwabla Pomegbe, Godwin Adzakpah, Nathan Kumasenu Mensah, Joseph Owusu-Marfo, Zenobia Wooduwa Asmah, Gloria Ekua Tawiah, Grace Amoah-Anomah

**Affiliations:** 1 Department of Health Information Management, School of Allied Health Sciences, University Post Office, University of Cape Coast, Cape Coast, Ghana; 2 Department of Marketing Studies, Cape Coast Technical University, Cape Coast, Ghana; 3 Department of Epidemiology, Biostatistics and Disease Control, University for Development Studies, Tamale, Ghana; 4 Ghana Education Service, Kwahu West Municipality, Nkawkaw, Ghana; Universiti Teknologi MARA, MALAYSIA

## Abstract

The coronavirus (COVID-19) pandemic has impacted various institutions significantly, including education. Although several studies have explored the transmission of the COVID-19 virus among humans health, few have investigated its impact on tertiary education in Ghana. This study, therefore, aimed to unmask the effects of COVID-19 on the academic performance of tertiary students in Ghana, specifically at the University of Cape Coast (UCC). Stratified and convenience sampling techniques were employed to select respondents from the College of Health and Allied Sciences at UCC for this study. Based on Krejcie and Morgan’s table for determining sample sizes for a large population in a study, a sample size of 531 was studied. Questionnaires were created and distributed to respondents from various departments to solicit their views. COVID-19 has significantly affected the academic performance of students at the University of Cape Coast, Ghana. A strong positive correlation was found between the positive impact of COVID-19 and academic performance (β = 3.385, *p* < 0.001). The study identified the absence of group discussions, poor internet networks, and other factors hurting students’ academic performance (β = -2.308, *p* < 0.001). Resources such as libraries, conducive environments in halls and hostels, and other factors also significantly influenced students’ academic performance (β = 2.941, *p* < 0.005). The study’s findings suggest that virtual learning platforms, adequate learning infrastructures, and internet packages should be available to students to facilitate teaching and learning as the University prepares for future pandemics.

## Background

Pandemics occur when a disease outbreak affects a large portion of the population across a wide area. Some pandemics have been recorded since 3000 B.C., including prehistoric instances [1]. The earliest pandemic recorded occurred during the Peloponnesian War; in 430 B.C., the plague of Athens also occurred [2]. The Athens epidemic struck soon after the battle of Athens and Sparta started, killing up to two-thirds of the populace. Between 1889 and 1890, the flu epidemic was another significant pandemic that claimed millions of lives and peaked in weeks [2]. It is surprising that the AIDS pandemic still affects up to 40 million people today, even though it was first identified in 1981 and is estimated to have killed 35 million people [1]. The most recent international health emergency is the coronavirus pandemic (COVID-19).

COVID-19 is predominantly comprised of viruses that typically originated in animals. Scientists refer to these groups of viruses as zoonotic, indicating a transmission from animals to humans [3]. The spread of COVID-19 among the human population occurs when an individual comes into contact with the secretions from an infected person or contact with infected surfaces [3]. A sore throat, fever, cough, and other symptoms are some COVID-19 markers. The COVID-19 pandemic was officially announced by the World Health Organization (WHO) in 2020 [4], following its initial detection in China-Wuhan in 2019. Due to COVID-19, "new norms" now include wearing nasal masks and isolating oneself from others [5]. As a result of these restrictions, the country’s economy suffered greatly, impacting both the health and education sectors. The government enacted measures in education to ensure that teaching and learning continues. Nonetheless, most of these proved futile because of unsuitable home learning environments, lack of technology and infrastructure, inaccessible learning materials and the inability of stakeholders to develop an appropriate curriculum to support teaching and learning. This situation resulted in setbacks in the education system. The pandemic affected many students transitioning to higher education or leaving school for career opportunities. The school administration was unable to remunerate their employees due to a lack of generated income. Several bus drivers responsible for transporting students to school did not get their wages, several stationery shops were forced to shut down, and a number of students were involved in malicious acts within their dormitories. This situation created significant financial and emotional instability for both parents and students. Based, on the reasons stated above, this current study examines how the coronavirus pandemic affected students academic performance the University of Cape Coast in Ghana. The study dramatically emphasizes the teaching and learning methods during the pandemic, socioeconomic factors influencing education, student challenges, and stakeholders’ readiness for imminent pandemics in tertiary education.

### A shift from traditional face-to-face learning

Africa faces diverse challenges, and improving the educational standards in Ghana is no exception. Education is an essential government activity; ignoring it is risky for the country [6]. According to [7], the first COVID-19 cases in Africa were reported in February 2020, following which the disease spread across the continent. As the number of cases increased, most governments moved quickly to manage and contain the pandemic. The Ministry of Health responded by closing schools as a safety measure. The study of [8] depicts that, while school closures were beneficial to the safety of students and teachers, reference [6] also postulates that no plans were made to replace traditional face-to-face classroom learning, which proved impractical for students’ ongoing education at home. Reference [8] inferred that even though the government launched some educational programs on television and radio for preparations towards examinations, teachers and students had no teaching and learning experiences outside the classroom. Universities had to resort to these techniques to ensure that continuation of distance teaching and learning becomes possible. Suitable study materials and systems were designed for the students to turn in tasks for assessment and monitoring progress like the lecturer’s assessment [9]. The University of Cape Coast uitilized online platforms such as Moodle Application, Google Meet, Google Classroom and Zoom Application for teaching and learning, although there were some interferences with their use. These systems for teaching and learning were only accessible to students with internet connections [10]. As a result, achieving effective teaching and learning with these systems approach was problematic. The approach was ineffective due to an unsuitable home learning environment, lack of technology and infrastructure, inaccessible learning materials and inability of stakeholders to develop an appropriate curriculum to support teaching and learning. To justify this study, the researchers envision COVID-19’s impact on the student’s academic performance at UCC. The researchers conceptualized a framework based on the constructs of items from the subject matter and proposed the following hypothesis;

Hypothesis 1: COVID-19 will positively impact students’ academic performance at UCC.
Hypothesis 2: COVID-19 will negatively impact students’ academic performance at UCC.
Hypothesis 3: adequate educational resources will influence students’ academic performance at UCC.

## Methods

### Study site description and respondents

This study was conducted at the University of Cape Coast (Ghana), established in October 1962. The University is currently the first University in Ghana and West Africa, respectively, 4^th^ best University in Africa and 24^th^ best University globally for research influence based on the Times Higher Education World University Rankings in 2023. The University runs programs for regular, sandwich, and distance learning students in both undergraduate and postgraduate programs. The initial student population of 155 in 1963 has grown to 74,720 in the 2021/2022 academic year. The College of Health and Allied Sciences under the School of Allied Health Sciences in the University runs 13 programs. These programs include Health Information Management (HIM), Medical Laboratory Technology (Med lab), Clinical Nutrition and Dietetics (CND), and Medical Imaging and Sonography (Med IS), just to mention a few. Due to the large student population sizes, the researchers focused on the students from the department mentioned above, considering time limitations, resource availability, and the high cost of conducting research in the entire college. This research received no specific grant from funding agencies in the public, commercial, or not-for-profit sectors.

### Study design, respondents and sampling

The study used a cross-sectional design to select respondents in the selected departments from August 9 to December 30, 2022. Stratified and convenience sampling methods were utilized to select the most available respondents from the large and dispersed student body of all 2021/2022 academic year students of HIM, CND, Med Lab, and Med IS in levels 300 and 400. However, level 100 and 200 students in the 2021/2022 academic year were excluded due to their non-enrollment at the University during Ghana’s COVID-19 lockdowns and hot periods. Before delivering questionnaires, respondents were informed of the research purpose and provided informed consent. Respondents took the survey before lectures. Researchers followed all COVID-19 requirements. Krejcie and Morgan’s sampling theory was used to select these respondents because researchers’ limited access to the entire population of interest required inferences from a representative sample.

Based on Krejcie and Morgan’s formula:

$$n=\frac{X^{2}\;NP\;(1-P)}{\left(d^{2}\;(N-1)+X^{2}\;P(1-P)\right)}$$

Where n = required sample size, N = population size, P = the population proportion (assume to be 0.5 since this would provide the maximum sample size), X^2^ = table value of chi-square, d = degree of accuracy expressed as a proportion (0.05). With a total population of 2001, a confidence interval of 95% gives a degree of accuracy of 0.05, and a degree of freedom of 1 provides a Chi-Square with a table value of 3.841.


$$n=\frac{3.841\times 2001\times 0.5\;(1-0.5)}{\left(0.05^{2}\;(2001-1)+3.841\times 0.5(1-0.5)\right)}$$

Therefore, from the Krejcie and Morgan table, a minimum sample size of 322 was expected.

### Ethics approval and consent to participate

This study was conducted with the approval of the Ethical Review Committee of the University of Cape Coast Institutional Review Board (approval number UCCIRB/CHAS/2022/145). A written informed consent was obtained from the study respondents after informing the aim of the study. Respondents reviewed consent forms a week before data collection.

## Data analysis and results

The data collected were analyzed using SPSS version 26 and Analysis of Moment of Structures (AMOS) Version 26. Descriptive and inferential statistical analysis was done to generate the necessary charts. Correlation and other studies were performed to establish the statistical significance of the appropriate variables of interest. In addition, a correlation analysis was performed to assess the impact of COVID-19 on academic performance using AMOS software. Table 1 shows the demographics of the respondents.

**Table 1:** Demographics of respondents.

Measure	Item	Frequency	Percentage (%)
Gender	Male	224	43.7
	Female	289	56.3
Age (years)	≥20	58	11.3
	21–24	332	64.7
	25–29	123	24.0
Department	HIM	129	25.1
	CND	130	25.3
	Med Lab	128	25.0
	Med IS	126	24.6
Level	300	219	42.7
	400	294	57.3

A total of 513 Allied Health students participated in the study. The Health Information Management Department had 129 (25.1%) responses, the Clinical Nutrition and Dietetics Department 130 (25.3%) responses, the Medical Laboratory Science Department 128(25.0%) responses and the Medical Imaging and Sonography Department126(24.6%) responses. Efforts were made to ensure a sampling procedure and distribution process of no bias. This accounts for the close gaps in percentages of reactions from these departments. Two hundred twenty-four (224) of the respondents were males (43.7%) and 289 were females (56.3%). A more significant percentage of respondents were females migrating from the Clinical Nutrition and Dietetics department, a female-dominated program at UCC. 219(42.7%) of the responses were students in level 300 and 294 from level 400 (57.3%). Most respondents were between the ages of 21 and 24 (332), with an overall rate of 64.7%. 123 of the respondents were between the ages of 25–29 years, a total rate of 24.0%. Fifty-eight (58) respondents were 20 years or below, accounting for the lowest percentage of 11.3%. This low percentage could be attributed to the fact that in the Ghanaian tertiary population, most undergraduate students fall between the ages of 21 and 24. Just a few of the tertiary population fall below or equal to 20 years, and these populations are most likely to be students who were admitted to UCC immediately after their secondary school education.

### Measurement of Confirmatory Factor Analysis (CFA)

Items loading over the advised levels were utilized, and measurement constructs were assessed for validity and reliability [11]. As many scholars have recommended, we operationalized students’ academic achievement at UCC as a reflective construct [12–17]. The measurement items were evaluated using construct reliability, which indicates the consistency of the measurement; convergent validity, which demonstrates that items are statistically linked with the appropriate constructs; and discriminant validity, which illustrates that determinants were independent [11–17] (see Table 2). All constructs were considerably and critically assessed based on how well they fit each construct. The Cronbach’s alpha for each of the individual constructs ranged from 0.9040 to 0.8550, above the threshold of 0.70. The Average Variance Extracted (AVE), which ranged from 0.7250 to 0.6330, was higher than the suggested level of 0.5 for all reflective components, indicating that the convergent validity was planned. Table 2 displays the metrics and confirms factor analysis.

**Table 2:** Measurement and confirmatory factory analysis.

Construct	Items	Unstandardized estimate	standard Error	Critical Ratio	Standard factor loadings	p-value	Average Variance Extracted	Composite Reliability	Cronbach’s alpha
Postive Impact	POSIMP1	1	-	-	0.941				
	POSIMP2	1.010	0.032	31.843	0.930	***			
	POSIMP3	0.660	0.042	15.546	0.601	***	0.633	0.869	0.855
	POSIMP4	0.796	0.046	17.434	0.649	***			
Negative Impact	NEGIMP1	1	-	-	0.967				
	NEGIMP2	1.014	0.023	44.880	0.950	***			
	NEGIMP3	0.789	0.034	22.945	0.738	***	0.725	0.912	0.904
	NEGIMP4	0.877	0.040	21.733	0.717	***			
Adequate Resources	ORESOU1	1	-	-	0.936				
	ORESOU2	0.982	0.032	30.649	0.913	***			
	ORESOU3	0.756	0.041	18.580	0.680	***	0.654	0.881	0.866
	ORESOU4	0.893	0.050	17.964	0.665	***			
Academic Performance	ACAPERF1	1	-	-	0.934				
	ACAPERF2	0.992	0.034	28.853	0.904	***			
	ACAPERF3	0.676	0.044	15.246	0.598	***	0.634	0.870	0.858
	ACAPERF4	0.892	0.047	19.169	0.700	***			

***P<0.001

The measurement models for the validity and reliability components were estimated using the CFA [17]. Eight metrics were employed in this study to assess the CFA’s goodness of fit (see Table 3). Every measurement was within range, indicating that the model was suitable for estimation. The overall fit of the research model is displayed in Table 3.

**Table 3:** Overall fit of the model.

Model-fit index	Recommended valve	Score	Interpretation
Chi-square/degree of freedom (χ2/df)	≤3.00	2.091	Excellent
Goodness-of-Fit Index (GFI)	≥0.90	0.953	Excellent
Adjusted Goodness-of-Fit Index (AGFI)	≥0.90	0.972	Excellent
Non-Normed Fit Index (NNFI)	≥0.90	0.959	Excellent
Comparative Fit Index (CFI)	≥0.90	0.980	Excellent
Root Mean Square Residual (RMR)	≤0.08	0.037	Excellent
Tucker-Lewis Index (TLI)	≥0.90	0.976	Excellent
Root Mean Square Error of Approximation (RMSEA)	≤0.08	0.049	Excellent

The standardized regression and correlations were pasted into the statswiki master validity calculator [11] to obtain the construct reliability and convergent and discriminant validity. According to the results, construct reliability was more significant than 0.70, and Average Variance Extracted (AVE) values were more substantial than 0.50. The Fornell and Lacker criteria (Table 4) and the Heterotrait-Monotrait (HTMT) (Table 5) ratios of correlation were both used in the study to assess discriminant validity. Thus, the square root of AVE, which has greater values that connect correlation coefficients, is used to substitute the matrix diagonals of the correlation coefficients [12]. This demonstrates how different reflecting constructions are from one another. For the structural model, the measurements in this investigation provided an acceptable validation of discriminant validity, convergent validity, and unidimensionality [12–15]. Table 5 presents discriminant validity using the Heterotrait-monotrait (HTMT) correlation ratio. The investigation found no alerts for discriminant validity using a tight criterion of 0.850 and a 0.900 barrier for liberal discriminant validity [16].

**Table 4:** Fornell and lacker criterion (discriminant validity).

	CR	AVE	MSV	MaxR(H)	NEGIMP	ORESOU	ACAPERF	POSIMP
**NEGIMP**	0.912	0.725	0.007	0.963	**0.851**			
**ORESOU**	0.881	0.654	0.041	0.932	0.050	**0.809**		
**ACAPERF**	0.870	0.634	0.009	0.927	-0.024	0.063	**0.796**	
**POSIMP**	0.869	0.633	0.041	0.939	0.086†	0.202***	0.096*	**0.796**

† p < 0.100

* p < 0.050

** p < 0.010

*** p < 0.001

√AVE are bold and underlined

**Table 5:** HTMT analysis.

	NEGIMP	ORESOU	ACAPERF	POSIMP
**NEGIMP**				
**ORESOU**	0.026			
**ACAPERF**	0.008	0.094		
**POSIMP**	0.133	0.228	0.073	

† p < 0.100

* p < 0.050

** p < 0.010

*** p < 0.001

## Discussion

### Hypothesis testing

**Fig 1:**
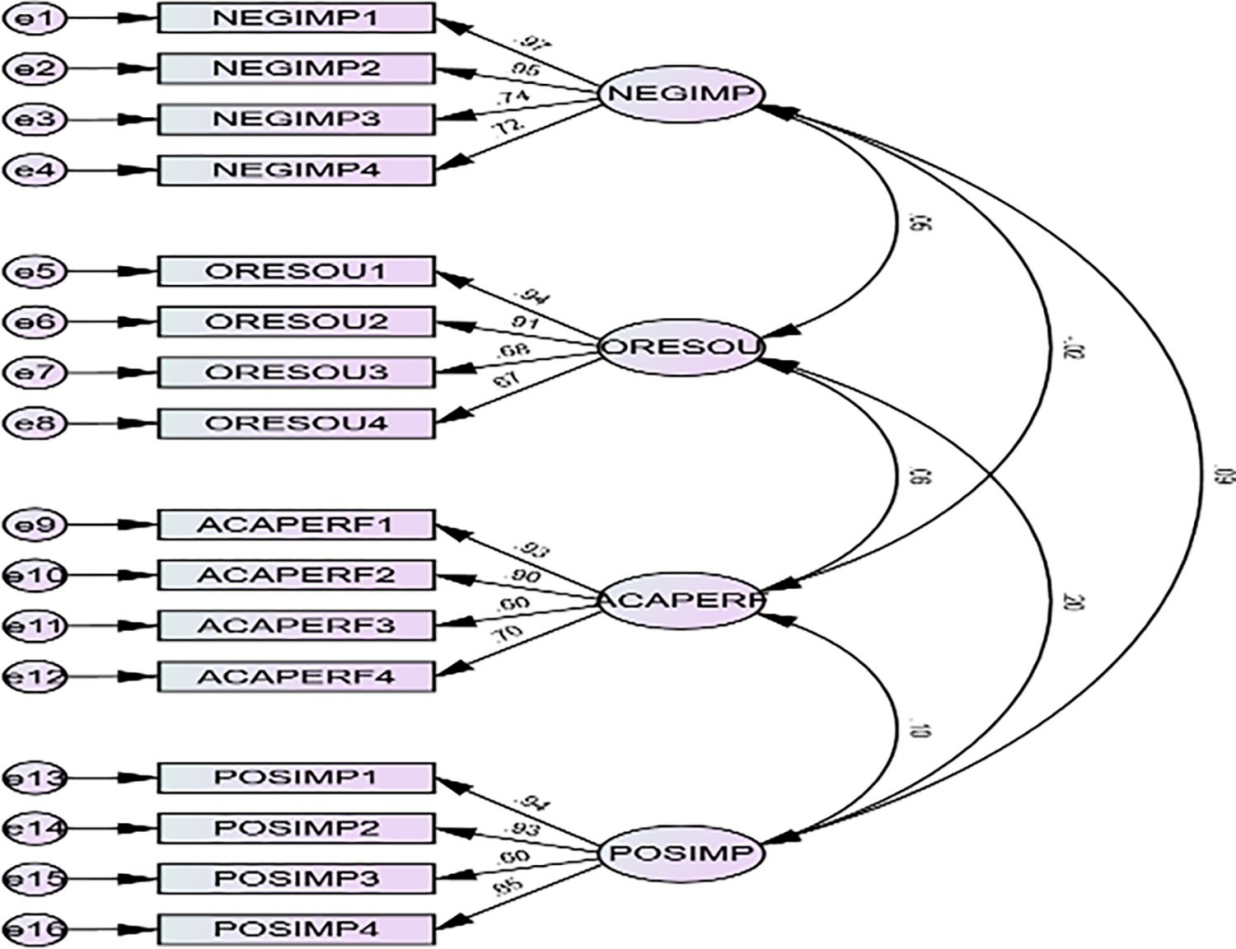


As presented in Table 3, the goodness-of-fit of the structural model was evaluated through a set of measures, which were found to be within acceptable limits. The results of the casual path diagram shown in Fig 1 supports the hypotheses and output from the AMOS software. The diagram demonstrated significant positive relationships for all hypotheses, albeit with varying significance levels. The constructs in the model are grounded in the empirical evidence produced by this study.

Hypothesis 1 posited a significant and positive impact of COVID-19 on academic performance. Results from the study (Table 6) showed a substantial relationship between COVID-19 and student academic performance, supporting the hypothesis (β = 3.385, p < 0.001). This finding aligns with research in Turkey [18], which found evidence of improved academic performance among science and social science students during the pandemic. The study also revealed strong teachers support for the transition to virtual learning and the implementation an attendance systems to encourage students participation. Meanwhile, students reported increased engagement and interaction during online lessons through virtual platforms like WeChat [19]. The inclusion of COVID-19-related course material added relevance to students’ studies, as noted by [20] in his assessment of veterinary medical students during the pandemic.

**Table 6:** Structural model results.

Hypothesis	Path	Standardized Estimate	Standard Error	Critical Ratio	Z-Value	Findings
H1	POSIMP -> ACAPERF	0.088	0.026	3.385	***	Supported
H2	NEGIMP -> ACAPERF	-0.030	0.013	-2.308	**	Supported
H3	ORESOU -> ACAPERF	0.050	0.017	2.941	**	Supported

t0.05 = 1.960 * t0.01 = 2.576 ** t0.001 = 3.29***

Contrary to this evidence, the results of [21] indicated that the pandemic had no significant effect on academic performance and that pre-pandemic performance levels were maintained. Our study found that students with high GPAs before COVID-19 demonstrated improved performance, while students with low GPAs showed decreased performance. We attribute these outcomes to the successful implementation of new learning approaches, the shift to virtual platforms, and students’ mastery of virtual tools such as Zoom, Google Classroom, and Moodle during the pandemic at the University of Cape Coast. The pandemic caused widespread disruption and hardship but brought positive developments, including integration of technology into the educational sector.

The COVID-19 pandemic has profoundly impacted the education system worldwide, including introducing virtual learning sessions. The current study supports the hypothesis of a negative and significant effect of COVID-19 on academic performance (β = -2.308, p < 0.000) as stated in hypothesis 2 (see Table 6). This finding is consistent with previous research, such as the work of [22], which investigated the satisfaction of Afghan students with online teaching and the impact of COVID-19 on their academic performance. The study found that most respondents preferred traditional face-to-face learning to virtual learning, and COVID-19 affected their ability to perform well in virtual course activities and assessments. Additionally, cultural perceptions and financial constraints, such as access to electronic devices, led to a lack of participation in online sessions, particularly among female Afghan students. The study also highlights the limitations of the information technology infrastructure in Afghanistan, leading to online classes being perceived as weak and ineffective. Studies by [23–25] have also shown the negative impact of COVID-19 on academic performance in Turkey, Mexico, and veterinary medical students, respectively. A common theme among these studies is the inadequate preparation for using information technology tools for virtual learning. The lack of training on effectively using these platforms made students dissatisfied with the online experience.

Furthermore, the absence of face-to-face interaction and group study sessions negatively impacted students’ academic performance. Implementing virtual learning and using information technology tools at the University of Cape Coast (UCC) was not accompanied by proper training sessions, leading to student dissatisfaction. This present study found that issues with network connectivity and the lack of face-to-face interaction negatively impacted the explanation of course materials by instructors’ and students’ understanding of the materials. Additionally, the absence of group study sessions had a detrimental effect on students’ academic performance. Results of the study showed that other educational resources significantly impact students’ academic performance in UCC, as measured by grades from quizzes and end-of-semester exams. A student’s grade reflects their performance and indicates that learning has taken place. An increase in performance capacity is evidence of learning, while a lack of growth may be attributed to factors such as infrastructure rather than the learning process itself.

In support of hypothesis 3, the present study found a significant impact of other educational resources on academic performance at the University of Cape Coast (UCC) with a beta value of 2.941 (p < 0.001) (See Table 6). This conclusion aligns with prior research by [26–29]. The study by [26] explored the effect of academic facilities on students’ academic achievement at Universiti Malaysia Kelantan (UMK) City Campus. The results revealed the importance of teaching aids and hostel facilities on students’ academic success. The cost of hostel rooms, the availability of amenities like water and electricity, the hostel’s location, and appearance were crucial factors influencing student satisfaction and their learning behaviours outside the lecture halls.

Similarly, Reference [27] highlighted that hostel amenities such as infirmaries, libraries, and study rooms can contribute to student comfort and influence their study patterns. Reference [28], a study of a private university in Kenya found that intrinsic factors such as motivation, the adoption of self-regulatory strategies, and self-efficacy significantly impact academic performance. The study suggested that good academic performance depends more on individual student factors like motivation and time management than external factors like campus resources and mentors. Finally, Reference [29] investigated educational facilities’ impact on business education students’ academic performance. The results showed that poor academic performance was associated with inadequate library resources. The study found a lack of current printed library materials, inadequate information technology tools, and insufficient internet connectivity to support academic work as the reasons behind the inadequate school library, contributing to the poor performance of students. The results of our investigation confirm the impact that social amenities, lecture materials, attendance, library resources, lecture hall quality, hostel conditions, and group studying have on students’ academic performance at the University of Cape Coast. These factors are crucial in shaping students’ educational outcomes and should be considered in future planning and decision-making processes.

## Study limitations

One of the limitations of this study is the use of a convenience sampling approach in selecting the respondents, which could have resulted in a selection bias and restricted the generalizability of the findings. Another limitation is that the respondents self-administered the questionnaires, which may have relied on their perceptions. To mitigate these limitations, future research should consider incorporating semi-structured interviews to provide in-depth insights or corroborate the results.

## Conclusion and recommendations

The present study aimed to examine the impact of the COVID-19 pandemic on students’ academic performance and learning at the University of Cape Coast. The research also aimed to determine the impact of educational facilities and the use of virtual platforms on students’ academic performance. The findings showed the pandemic’s positive and negative effects on students’ academic performance. The study found that mastery of virtual tools, such as the Moodle application, Google Classroom, and Zoom, positively impacted students’ learning activities during COVID-19. On the other hand, the absence of group discussions harmed students’ performance.

Furthermore, the study found that adequate educational resources, such as libraries, hostels, lecture halls, and proper lighting and acoustics, significantly contributed to students’ academic performance. The study highlights the crucial role of technology in education and the need for incorporating technological tools to sustain and continue learning outside the classroom. The study also revealed the benefits of virtual platforms, such as increased student participation and interaction in course discussions. Based on the findings, the study recommends that the university administration and government take steps to provide students with electronic gadgets, such as laptops, and make payment options accessible. The administration should also ensure the provision of Wi-Fi packages and strong internet connections for students to facilitate the use of virtual platforms. To ensure the effective use of virtual platforms for distance teaching and learning, lecturers and students should be provided with adequate training. Additionally, the study suggests that e-learning portal developers incorporate real-time interaction and feedback sections to allow for student-lecturer interaction and coursework criticism.

## Supporting information

S1 Data (SAV)

S1 Questionnaire (DOCX)
