# Supplementary material for: Unmasking the coronavirus pandemic impact on students’ academic performance (University of Cape Coast—Ghana)
Source: PLOS Glob Public Health. 2024 Jul 16;4(7):e0003409. doi: 10.1371/journal.pgph.0003409 (PMC11251575; doi:10.1371/journal.pgph.0003409)
Supplement: S1 Questionnaire — (DOCX) [file pgph.0003409.s002.docx]

**UNIVERSITY OF CAPE COAST**

**COLLEGE OF HEALTH AND ALLIED SCHIENCES**

**SCHOOL OF ALLIED HEALTH SCIENCES**

**DEPARTMENT OF HEALTH INFORMATION MANAGEMENT**

QUESTIONNAIRE TO DOCUMENT THE IMPACT OF CORONAVIRUS PANDEMIC ON STUDENTS’ ACADEMIC PERFORMANCE IN TERTIARY SCHOOLS (UNIVERSITY OF CAPE COAST – GHANA)

Dear respondent,

This questionnaire seeks to analyze the impact of Coronavirus pandemic on students’ academic performance in tertiary schools (University of Cape Coast – Ghana)

Please be informed that this study is purely academic and that all information obtained shall be kept with outmost confidentiality. The outcome of this research may be used for academic and general purposes such as research reports, conference papers or books. You are free to withdraw from this research at any time.

Thank you in advance for your co-operation**.**

**PART I: SOCIO-DEMOGRAPHIC CHARACTERISTICS OF PARTICIPANTS**

Please tick (✔) the response applicable to you.

| 1. **Level:** 300 400 |
| --- |
| 1. **Age group:** 1. 20yrs and below 2. 21– 24yrs 3. 25– 29 yrs 4. 30 and above yrs |
| 1. **Gender:** 1. Male 2. Female |
| 1. **Department/unit _________________________________________** |

**PART II and III:** What factors (Resources and academic) contribute to good academic performance for students in UCC?

Please tick (✔) the response as applicable to you.

| Statement | STRONGLY AGREE | AGREE | NEUTRAL | DISAGREE | STRONGLY DISAGREE |
| --- | --- | --- | --- | --- | --- |
|  | 1 | 2 | 3 | 4 | 5 |
| 1. Do you accept that library resources contribute to good academic performance? |  |  |  |  |  |
| 1. Are there course materials available in the university library to aid in your studies? |  |  |  |  |  |
| 1. Do environmental factors such as lighting and noise affect academic performance? |  |  |  |  |  |
| 1. Does group studies/or individual studies contribute to good academic performance? |  |  |  |  |  |
| 1. Does participation in lectures contributes to good academic performance? |  |  |  |  |  |
| 1. Does frequent attendance to lectures promote good academic performance? |  |  |  |  |  |
| 1. Do lecturers provide students with course materials? |  |  |  |  |  |
| 1. Do the course materials have great impact on your academic performance? |  |  |  |  |  |

**PART IV**: What are the positive effects of COVID-19 on the performance and learning activities of students in UCC?

|  | STRONGLY AGREE | AGREE | NEUTRAL | DISAGREE | STRONGLY DISAGREE |
| --- | --- | --- | --- | --- | --- |
|  | 1 | 2 | 3 | 4 | 5 |
| 1.Were new approaches to studies introduced by your lecturers during COVID-19 break? |  |  |  |  |  |
| 2. Were you able to master the use of new technological skill during COVID-19? |  |  |  |  |  |
| 3.Did the COVID-19 pandemic provide you with a new and better approach of studying? |  |  |  |  |  |
| 4.Online learning tools such as zoom, google meet, skype, whatsapp, telegram and others were used during distance learning. |  |  |  |  |  |
|  |  |  |  |  |  |

**PART V**: What negative impacts has COVID-19 had on the learning and performance of students in UCC?

|  | STRONGLY AGREE | AGREE | NEUTRAL | DISAGREE | STRONGLY DISAGREE |
| --- | --- | --- | --- | --- | --- |
|  | 1 | 2 | 3 | 4 | 5 |
| 1. Were course materials thoroughly explained by lecturers during all online classes? |  |  |  |  |  |
| 2. Did online classes provide you with better understanding to course work than face-to-face lectures? |  |  |  |  |  |
| 3. Did the absence of group meetings to discuss course materials with colleagues affected your studies? |  |  |  |  |  |
| 4. Was the external environment and home environment distracting during online classes? |  |  |  |  |  |
